# Lactoferrin-Loaded Liposomal Nanoparticles: Enhanced Intestinal Stability and Bioactivity for Mitigating Radiation-Induced Intestinal Injury

**DOI:** 10.3390/foods14193410

**Published:** 2025-10-02

**Authors:** Yingying Lin, Rui Ding, Yuning Zhang, Yimeng Wang, Sijia Song, Huiyuan Guo

**Affiliations:** 1 Key Laboratory of Functional Dairy, Department of Nutrition and Health, China Agricultural University, NO. 10 Tianxiu Road, Haidian, Beijing 100191, China; linyingying@cau.edu.cn (Y.L.); dingrui990606@163.com (R.D.); 18537927291@163.com (Y.W.); songsijia1996@cau.edu.cn (S.S.); 2 Key Laboratory of Precision Nutrition and Food Quality, College of Food Science and Nutritional Engineering, China Agricultural University, Beijing 100083, China; bs20243060693@cau.edu.cn; 3 Food Laboratory of Zhongyuan, Luohe 462300, China

**Keywords:** lactoferrin, liposomal nanoparticles, targeted delivery, radiation-induced intestinal injury

## Abstract

Radiation-induced intestinal injury (RIII), a severe complication of abdominopelvic radiotherapy, causes intestinal ischemia, ulcers, and necrosis, severely impacting patients’ quality of life. Currently, effective treatments are limited, and a specific cure remains elusive. Our previous research showed that lactoferrin (LF) can promote intestinal stem cell (ISC) proliferation and tissue repair; however, its oral administration is limited by rapid degradation in the gastric environment. In this study, we developed LF-loaded liposomal nanoparticles (Lip-LF) using a simple ethanol injection method. The optimal formulation (cholesterol/egg yolk lecithin ratio 2:8, LF concentration 12.5 mg/mL) achieved a drug-loading capacity of 6.8% and a narrow size distribution (0.2 < PDI < 0.4). In vitro experiments demonstrated that Lip-LF protected LF from pepsin degradation in simulated gastric fluid (SGF), retaining over 80% integrity after 120 min, while releasing in simulated intestinal fluid (SIF). In vivo imaging revealed prolonged gastrointestinal retention of Lip-LF compared to free LF. In a murine model of RIII (12 Gy whole-body irradiation), Lip-LF significantly restored villus counts, increased crypt height, and promoted goblet-cell regeneration. Immunohistochemical and qPCR analyses revealed enhanced ISCs proliferation and upregulation of repair-associated genes, including *Pcna* and *Olfm4*. These findings demonstrate that Lip-LF protects LF from gastric degradation and enhances its targeted delivery to the intestine, improving its therapeutic efficacy in repairing RIII. Lip-LF thus offers a promising strategy for managing RIII.

## 1. Introduction

Radiation-induced intestinal injury (RIII) remains one of the most frequent and dose-limiting adverse effects of abdominal or pelvic radiotherapy, whose key mechanisms include IEC (IEC) damage, inflammation, and gut microbiota disruption, often manifesting as diarrhea, malabsorption, and intestinal bleeding [1]. These symptoms not only impair the quality of life but also force treatment interruptions, thereby compromising oncological outcomes [2,3]. At present, no targeted pharmacological therapy is available, and management is restricted to symptomatic support such as antidiarrheals, fluid resuscitation, and parenteral nutrition. The need for mild yet effective nutritional interventions to strengthen the intestinal mucosa against radiation damage is increasing.

Lactoferrin (LF), an 80 kDa iron-binding glycoprotein abundantly present in mammalian milk and exocrine secretions, exerts pleiotropic biological activities including antimicrobial, anti-inflammatory, antioxidant, and immunomodulatory effects [4]. In addition, LF exerts beneficial effects on intestinal health by enhancing the intestinal epithelial barrier, modulating inflammation, and optimizing the gut microbiota [5,6,7]. LF upregulates the expression of tight-junction proteins, thereby reducing pathogen permeability and reinforcing the intestinal barrier [5,6]. In animal models, LF has been shown to mitigate the severity of necrotizing enterocolitis (NEC) by accelerating crypt regeneration and restoring epithelial integrity [8]. Clinical trials have demonstrated that LF supplementation reduces the incidence and duration of diarrhea, improves hemoglobin levels in anemic infants, and halves the incidence and mortality of stage ≥2 NEC in very-low-birth-weight neonates [9,10]. Additionally, LF modulates the gut microbiota by promoting the growth of beneficial bacteria and inhibiting pathogenic bacteria, thereby improving intestinal morphology and function [11,12]. These attributes render LF an attractive candidate for mitigating RIII. However, native LF is highly susceptible to peptic and tryptic degradation in the gastric milieu, resulting in limited bioavailability of intact protein to the small intestine, where its protective actions are most needed.

To enhance its stability and bioavailability in the gastrointestinal tract, various stabilization techniques have been developed, including chemical modifications (e.g., PEGylation) and encapsulation methods (e.g., liposomes or solid lipid particles) [13,14,15]. Among these, liposomal encapsulation has emerged as a promising solution due to its ability to protect bioactive proteins from enzymatic degradation while enhancing intestinal absorption. Liposomes, with their phospholipid bilayer structure, not only improve the stability and solubility of LF but also prolong intestinal residence time through mucoadhesion [16,17,18]. Researchers have utilized naturally abundant lipids and sterols to develop liposomes, which have demonstrated improved resistance to pepsin digestion and protective effects against hepatic injury in animal models [15,19].

While conventional liposome preparation methods face challenges in scalability and food-grade applicability, the ethanol injection technique offers distinct advantages [20]. This mild and scalable method enables precise control over liposome formation while shielding the cargo from peptic and tryptic degradation and enabling targeted release in the small intestine [20,21]. Importantly, it allows the use of food-grade lipids (e.g., egg yolk lecithin) to develop stable LF-loaded liposomes that resist gastric degradation and enable targeted intestinal release. Building on these advantages, we propose the ethanol injection method for fabricating food-compatible LF liposomes. This approach addresses critical requirements for food applications: Generally Recognized as Safe (GRAS) material compliance, high encapsulation efficiency, and industrial scalability, while overcoming the current limitations such as phospholipid oxidation and LF leakage during storage. The resulting formulation is expected to significantly enhance LF’s therapeutic efficacy against intestinal disorders while meeting stringent food safety standards.

In this study, we designed and developed LF-loaded liposomal nanoparticles (LF-Lip) to enhance the efficacy of LF in mitigating RIII. LF was encapsulated within liposomes using the ethanol injection method. The physicochemical properties of LF-Lip, including particle size distribution, morphology, and stability, were thoroughly characterized, and the biodistribution and mucosal retention of LF-Lip were also evaluated. Furthermore, in a murine model of RIII, the functions and mechanisms of LF-Lip in mitigating RIII were explored, with a particular focus on its effects on intestinal epithelial structure, stem cell survival, and epithelial cell proliferation and differentiation. These experimental results suggest that LF-Lip could serve as a promising nutritional intervention for the management of RIII.

## 2. Materials and Methods

### 2.1. Materials

Egg yolk phosphatidylcholine (EYPC, ≥98%) was purchased from Solarbio (Beijing, China), and cholesterol (Chol, ≥99%) was purchased from Sigma-Aldrich (St. Louis, MO, USA). Bovine lactoferrin (LF, ≥95% purity, 80 kDa) was obtained from Fonterra Ltd. (Auckland, New Zealand). Cy5-NHS ester was from MedChemExpress (Monmouth Junction, NJ, USA). BCA protein assay kit, protease/phosphatase inhibitor cocktails, RIPA buffer, and PMSF were supplied by Beyotime (Shanghai, China). Simulated gastric fluid (SGF) and simulated intestinal fluid (SIF) components (pepsin, pancreatin, and bile salts) were prepared according to the INFOGEST consensus protocol [22]. All the other reagents were of analytical grade and used as received.

### 2.2. Preparation of Lip-LF

**Figure 1:**
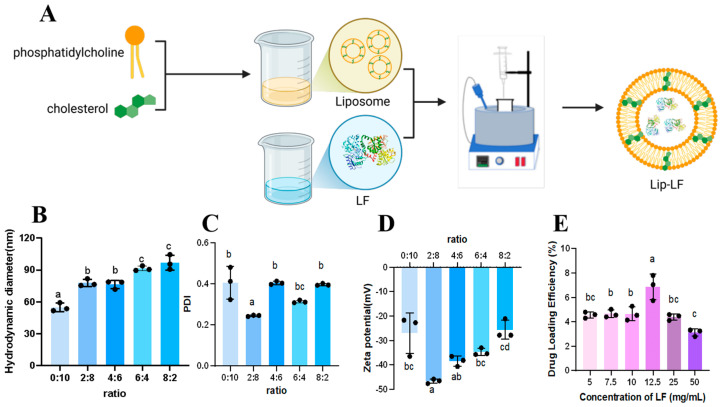
Influence of lipid composition on particle size and PDI. (**A**) Schematic illustration of the preparation process of Lip-LF. (**B**–**D**) Effect of different yolk lecithin cholesterol ratios on particle size (**B**), PDI value (**C**), and Zeta potential (**D**) of liposomes. PDI: Polydispersity Index, a key parameter reflecting particle size distribution uniformity. (**E**) Effect of different LF concentrations on drug loading efficiency of liposomes. Data are shown as mean ± SD, *n* = 3. The significance of differences among multiple groups was evaluated using one-way ANOVA, followed by Duncan’s post hoc test. a, b, and c represent significant differences (*p* < 0.05).

Lip-LF were fabricated by a modified ethanol-injection method [20]. Briefly, Chol and EYPC at mass ratios of 8:2, 6:4, 4:6, and 2:8 (totaling 100 mg) were dissolved in 10 mL of absolute ethanol and sonicated for 60 min. One mL of the organic phase was rapidly injected into five mL of aqueous phase containing LF (12.5 mg mL^−1^ unless otherwise specified) under magnetic stirring (600 rpm, 25 °C). Ethanol was allowed to evaporate for 2 h to yield Lip-LF. The 10:0 ratio (Chol only) was excluded because phospholipid is indispensable for bilayer formation. Schematic preparation is depicted in Figure 1A.

### 2.3. Drug Loading Determination

The optimal Chol: EYPC ratio (2:8, *w*/*w*) was used for drug-loading studies. LF was dissolved in the aqueous phase at 5, 7.5, 10, 12.5, 25, or 50 mg mL^−1^. After liposome formation, free and encapsulated LF were separated by ultracentrifugation (4 °C, 12,000 rpm, 1 h). The pellet was lysed with RIPA buffer supplemented with 1% PMSF, 1% protease inhibitor, and 1% phosphatase inhibitor (30 min on ice). Lysis was completed by sonication (25% power, 9 s on/9 s off, 3 cycles) followed by centrifugation (12,000 rpm, 25 min, 4 °C). Protein concentration in the supernatant was quantified by BCA assay using a bovine serum albumin standard. Loading efficiency (LE) was calculated as follows: LE (%) = (Wt − Wf)/Wlip × 100, where Wt, Wf, and Wlip represent total LF added, unencapsulated LF, and total liposome weight, respectively.

### 2.4. Physicochemical Characterization

#### 2.4.1. Particle Size and Polydispersity Index (PDI)

Hydrodynamic diameter and PDI were measured by dynamic light scattering (DLS) using a Malvern Zetasizer Nano ZS90 (Malvern, UK) at 25 °C with a 90° scattering angle. The samples (1 mL) were equilibrated for 120 s and analyzed in triplicate.

#### 2.4.2. Morphology

A 10 μL aliquot of Lip-LF was deposited on a carbon-coated 200-mesh copper grid, allowed to adsorb for 10 min, gently blotted, and rinsed with ultrapure water. After air-drying for 30 min, the samples were imaged by transmission electron microscopy (TEM, JEM-2100, JEOL, Tokyo, Japan) at 200 kV.

#### 2.4.3. Zeta Potential

Zeta potential was determined by laser Doppler electrophoresis using the same instrument. Liposomes were diluted in ultrapure water (900 μL) and measured in folded capillary cells at 25 °C (*n* = 3).

#### 2.4.4. Storage Stability

Freshly prepared Lip-LF were stored at 4 °C for 6 days. Particle size and PDI were monitored on days 1, 2, 3, 4, 5, and 6.

### 2.5. In Vitro Digestive Stability

#### 2.5.1. Simulated Gastrointestinal Digestion

Digestion experiments were performed following the INFOGEST protocol [22] to simulate the gastrointestinal digestion process. Lip-LF was mixed 1:1 (*v*/*v*) with SGF (pH 3.0) containing pepsin (2000 U mL^−1^) and incubated at 37 °C with shaking at 180 rpm for 2 h. The gastric digestion was terminated by raising the pH to 7.0. Subsequently, an equal volume of SIF (pH 7.0) containing bile salts (10 mM) and pancreatin (100 U mL^−1^ trypsin activity) was added and incubated for another 2 h under identical conditions. Aliquots were withdrawn at 30, 60, and 120 min; intestinal digestion was stopped by heating at 95 °C for 10 min.

#### 2.5.2. SDS-PAGE Analysis

The protein samples were adjusted to equal concentrations, mixed with a 5× SDS-PAGE loading buffer, and heated at 100 °C for 10 min to achieve denaturation. The samples were then subjected to electrophoresis on a 10% SDS-PAGE gel, initially run at 80 V and then increased to 120 V to complete the separation. Following electrophoresis, the gels were stained with Coomassie Brilliant Blue R-250 for 30 min, followed by destaining until the protein bands were clearly visible. Gel images were captured using a gel documentation system.

#### 2.5.3. Confocal Laser Scanning Microscopy (CLSM)

For microscopic analysis, 30 μL of the digested samples were mounted onto glass slides with anti-fade mounting medium. The samples were observed under a Zeiss LSM 880 microscope (Carl Zeiss, Jena, Germany) using Cy5 fluorescence settings to visualize the distribution and integrity of the components within the samples.

### 2.6. In Vivo Gastrointestinal Distribution

#### 2.6.1. Fluorescent Labeling of LF

LF (12.5 mg mL^−1^) was conjugated with Cy5-NHS (30 μL) under gentle rotation (4 h, dark). Free dye was removed by ultrafiltration (10 kDa MWCO, 4 °C, 4000 g, 5 cycles). Labeled LF was reconstituted to 12.5 mg mL^−1^ before liposome preparation.

#### 2.6.2. Animal Imaging

Male C57BL/6J mice (8 weeks, SPF) were fasted overnight and randomly divided into two groups (*n* = 12). The mice received 200 μL of either free Cy5-LF or Cy5-Lip-LF (50 mg kg^−1^) via gavage. At 0.5, 1, 2, and 4 h post-administration, the animals were euthanized (*n* = 3), and the entire gastrointestinal tract was excised for in situ imaging using an IVIS Spectrum system (PerkinElmer, Shelton, CT, USA).

### 2.7. Animal Ethics and Experimental Design

#### 2.7.1. Animals and Husbandry

C57BL/6J mice (8 weeks, male) were purchased from SPF Biotechnology (Beijing, China) and acclimatized for 7 days under controlled conditions (20–22 °C, 50–60% humidity, 12 h light/dark cycle) with ad libitum access to standard chow and water. All the procedures were approved by the Institutional Animal Care and Use Committee of China Agricultural University (approval No. AW72205202-5-3).

#### 2.7.2. Radiation Enteropathy Model and Treatments

Following acclimatization, the mice were randomized into four groups (*n* = 6): (i) control (con, water), (ii) empty liposome (Lip), (iii) Free LF (LF, 50 mg kg^−1^), and (iv) Lip-LF (50 mg kg^−1^). The treatments were administered daily by gavage for 7 consecutive days. On day 4, the mice received total-body γ-irradiation (^60^Co source, 12 Gy) at Peking University. Day 3.5 post-irradiation (regenerative phase) was selected as the sampling time-point. Body weight, and small-intestinal and colonic lengths and weights were recorded. Duodenum, jejunum, and ileum were collected for histology; jejunum was also used for molecular analyses.

#### 2.7.3. Systemic Toxicity Evaluation

A separate cohort (*n* = 3) received identical gavage for 7 days. On day 8, the animals were euthanized, and major metabolic organs (spleen, lung, kidney, and liver) were harvested for gross and histopathological evaluation.

### 2.8. Hematoxylin-Eosin (H&E)

Fresh intestinal tissues were fixed in 4% paraformaldehyde (PFA) for 24 h, followed by gradient ethanol dehydration. The dehydrated tissues were then embedded in paraffin. Subsequently, the tissue was sectioned into 4 um slices. The tissue sections were deparaffinized in xylene and rehydrated through a graded ethanol series, after which the staining procedure was initiated. Then, the deparaffinized tissue sections were stained with hematoxylin for 30 s and eosin for 10 s, followed by dehydration and mounting in neutral resin for microscopic examination.

### 2.9. Histological Injury Score

Intestinal epithelial injury was assessed according to Chiu’s classification method [23]. Grade 0 indicates normal intestinal mucosal villi. Grade 1 is characterized by capillary hyperemia and cystic gaps beneath the epithelium at the villus apex. Grade 2 features enlarged cystic gaps beneath the epithelium, edema extending into the lamina propria, and dilation of the central lacteal vessels. Grade 3 involves degeneration and necrosis of intestinal epithelial cells (IECs), severe edema in the lamina propria, and occasional villus tip abscission. Grade 4 includes degeneration, necrosis, and exfoliation of IECs, capillary hyperemia and dilation, exposure of the lamina propria, and abscission in some villi. Grade 5 is marked by bleeding, ulceration, disintegration of the lamina propria, and complete villus abscission.

### 2.10. Periodic Acid-Schiff (PAS) Staining

PAS staining was performed using a PAS stain kit (Leagene, #DG0005, Beijing, China) according to the manufacturer’s guidelines. In brief, the slides were subjected to the same hydration steps as those used in the H&E analysis until placement into distilled water, and the paraffin slides were then immersed in periodic acid solution for 8 min and subjected to one 3 min rinse in tap water and two 3 min rinses in distilled water. The slides were subsequently immersed in Schiff’s solution for 10 min and then rinsed for 10 min with tap water. Hematoxylin staining was then performed for 30 s to stain the nucleus. After another 10 min rinse in tap water, the slides were subjected to the same steps as those used in the H&E staining until placement in xylene and then mounted with neutral balsam, and examined under microscopy.

### 2.11. Immunohistochemistry Staining

Tissue sections were subjected to fixation and dewaxing according to the previously described H&E staining protocol. Antigen retrieval was subsequently performed by microwave heating in citrate buffer (pH = 6.0) for 30 min. After cooling to room temperature, endogenous peroxidase activity was blocked with 3% hydrogen peroxide solution, followed by 1 h incubation with blocking buffer at room temperature. Sections were then incubated with primary antibody against PCNA (1:200, sc-56, Santa Cruz Biotechnology, Inc., Dallas, TX, USA) at 4 °C overnight, followed by secondary antibody incubation at room temperature. Target proteins were visualized using diaminobenzidine tetrahydrochloride (DAB), with hematoxylin counterstaining. Finally, sections were dehydrated and mounted in neutral resin for microscopic examination.

### 2.12. Immunofluorescence Staining

The tissue sections were processed for fixation, dewaxing, and antigen retrieval according to the immunofluorescence staining protocol. After that, the sections were incubated with the primary antibody against Sox9 (1:2000, ab3697, Abcam, Waltham, MA, USA), followed by incubation with the secondary antibody. Cell nuclei were counterstained with DAPI working solution. Finally, the sections were then mounted with an anti-fade mounting medium to preserve fluorescence.

### 2.13. Quantitative Real-Time PCR (qPCR)

Total RNA was extracted from the jejunum using TRIzol reagent (Invitrogen, Carlsbad, CA, USA) and reverse-transcribed with PrimeScript RT Master Mix (Takara, Kusatsu, Japan). qPCR was performed on a CFX96 system (Bio-Rad, Hercules, CA, USA) using SYBR Green Master Mix. The primer sequences are listed in Table 1. Relative gene expression was calculated by the 2^−ΔΔCt^ method using β-actin as an internal control.

### 2.14. Statistical Analysis

Data are presented as mean ± SD. Statistical significance was determined by one-way ANOVA followed by Tukey’s post hoc test using GraphPad Prism 9.0. *p* < 0.05 was considered statistically significant.

## 3. Results

### 3.1. Optimization of Lip-LF Formulation

#### 3.1.1. Influence of Lipid Composition on Particle Size and PDI

Phospholipid/cholesterol ratio is a key determinant of liposomal physicochemical stability [24,25]. As illustrated in Figure 1B, increasing cholesterol content (Chol: EYPC from 0:10 to 8:2) resulted in a progressive enlargement of particle diameter. Concomitantly, the PDI, a key parameter reflecting particle size distribution uniformity, exhibited a biphasic trend. A higher PDI value indicates greater particle size variation and reduced stability during storage. The influence of the Chol/EYPC mass ratio on liposome size and PDI is evident in Figure 1. Notably, the 2:8 ratio achieved the lowest PDI value compared to other ratios (0:10, 4:6, 6:4, and 8:2), indicating a more uniform size distribution (Figure 1C). Additionally, the zeta potential (−46.63 ± 4.70), another stability indicator, was highest in absolute value for the 2:8 ratio, facilitating adsorption of positively charged LF (Figure 1D). Based on these results, the 2:8 ratio was chosen for liposome preparation.

#### 3.1.2. Drug-Loading Optimization

The drug loading capacity of liposomes is a critical parameter in drug delivery system design, as higher loading reduces dosage and costs while enhancing therapeutic efficacy [26]. However, increasing drug concentration to enhance loading capacity may destabilize liposomes, resulting in a decline in loading efficiency. In this study, we optimized the drug concentration in liposomes. As shown in Figure 1E, increasing LF concentration initially increased drug loading capacity but then decreased it. This may be due to enhanced interactions between LF and drug molecules at higher concentrations, causing aggregation or precipitation of the drug [27]. Liposomes with an LF concentration of 12.5 mg/mL exhibited the highest drug loading capacity. Therefore, the drug loading concentration of 12.5 mg/mL was established as the optimal level for liposomes.

### 3.2. Comprehensive Characterization of Lip-LF

**Figure 2:**
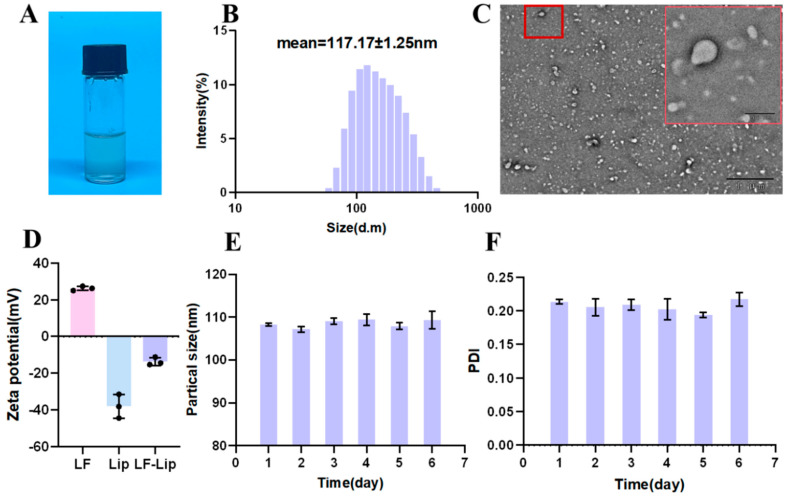
Physicochemical characterization of Lip-LF: (**A**) Physical image of Lip-LF. (**B**) Size distribution of Lip-LF. (**C**) Transmission electron microscopy of LF-Lip. (**D**) Zeta potential measurement of Lip, LF, and Lip-LF. (**E**,**F**) Changes in particle size (**E**) and PDI value (**F**) of Lip-LF after 6 days of storage. Data are shown as mean ± SD, *n* = 3.

Following the successful synthesis of Lip-LF, we systematically evaluated its physicochemical properties and structural integrity through multiple analytical techniques to confirm its suitability as a stable nanocarrier system. Lip-LF exhibited a clear and homogeneous liquid appearance with no evidence of precipitation, as observed in Figure 2A. TEM micrographs revealed uniformly spherical vesicles with a clear bilayer shell and an electron-lucent aqueous core (Figure 2C), consistent with the hydrodynamic diameter obtained by DLS (Figure 2B). The negative charge of Chol/EYPC liposomes allows for greater LF encapsulation via electrostatic attraction, but also causes LF adsorption on the liposome surface, leading to a higher zeta potential (−38.03 ± 5.30 mV) for LF-lip than for liposomes alone (Figure 2D). During 6-day storage at 4 °C, Lip-LF exhibited stable particle size and minimal PDI fluctuations (Figure 2E,F), reflecting excellent physical stability without aggregation or precipitation.

### 3.3. In Vitro Gastrointestinal Protection and Release

**Figure 3:**
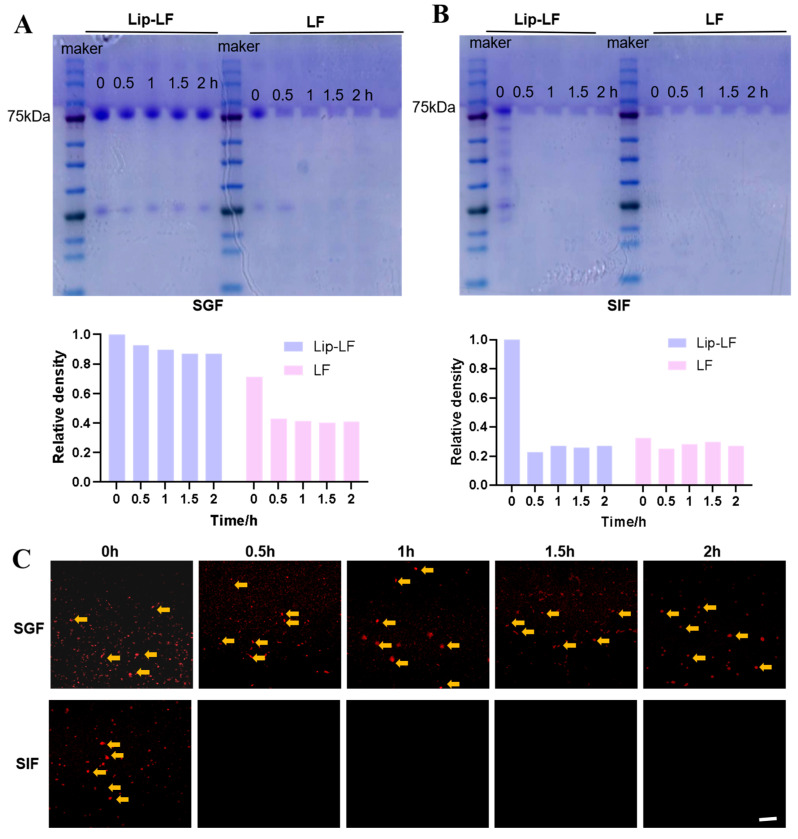
The stability and bioavailability of Lip-LF: (**A**,**B**) SDS-PAGE image of LF retention of Lip-LF at 0 h, 0.5 h, 1 h, 1.5 h, and 2 h after SGF (**A**) and SIF (**B**). (**C**) Confocal laser scanning micrographs of LF and Lip-LF at 0 h, 0.5 h, 1 h, 1.5 h, and 2 h after SGF and SIF treatment. LF was labeled with Cy5-NHS. SGF: simulated gastric digestive fluid; SIF: simulated intestinal digestive fluid.

To investigate the digestive characteristics of Lip-LF, this study evaluated their protective efficacy for LF using an in vitro gastrointestinal digestion model. Following 120 min simulated gastric digestion, Lip-LF retained 80.1% of intact LF, whereas free LF was completely hydrolyzed within 30 min (Figure 3A). The disappearance of the band after 30 min of intestinal digestion of Lip-LF confirms that Lip-LF can release LF in the intestine, where it is digested, degraded, and exerts its effects. CLSM imaging revealed persistent Cy5 fluorescence in Lip-LF during the gastric phase, which rapidly diminished upon transfer to intestinal fluid (Figure 3B), confirming pH-triggered release and proteolytic degradation. These data demonstrate that liposomal encapsulation effectively shields LF from gastric enzymes and ensures site-specific release in the intestine.

### 3.4. In Vivo Gastrointestinal Biodistribution

**Figure 4:**
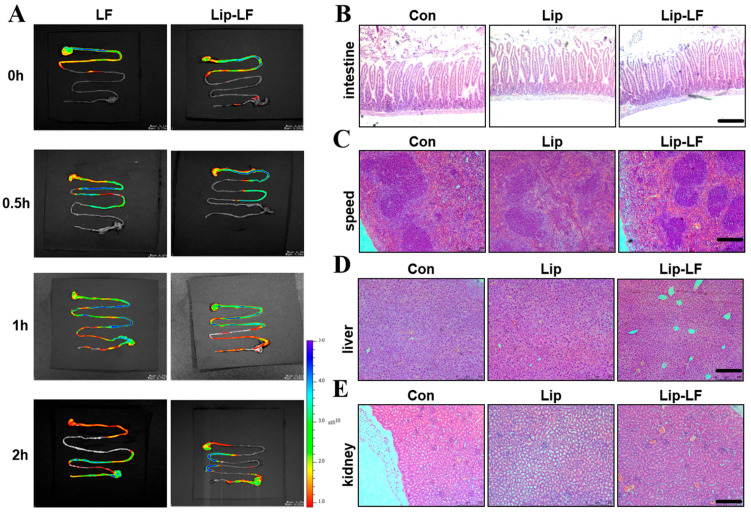
Distribution and histological analysis of Lip-LF in mice after oral gavage: (**A**) The figure illustrates the distribution of LF in the gastrointestinal tract after oral gavage of free LF and Lip-LF. (**B**–**E**) H&E staining of the small intestine (**B**), spleen (**C**), liver (**D**), and kidney (**E**) of mice after one week of different treatments by gavage (*n* = 3 mice/group, scale bar, 100 μm).

Cy5-labeled LF was administered to mice via gavage, and its digestion and distribution in the gastrointestinal tract were examined using a small animal fluorescence imaging system at 0, 30, 60, and 120 min post-administration. Real-time imaging showed markedly prolonged retention of Lip-LF within the small intestine compared to free LF (Figure 4A). After 2 h, Lip-LF were predominantly localized in the jejunum, whereas free LF had already transited to the cecum and colon. These findings indicate that liposomal encapsulation retards gastric emptying and enhances mucosal adhesion, thereby increasing local therapeutic exposure.

No macroscopic or histopathological abnormalities were observed in the liver, kidney, spleen, or lung after 7-day gavage of Lip-LF (Figure 4B–E). Intestinal villi remained intact, underscoring the biocompatibility of the ethanol-injection-derived liposomes.

Collectively, these results demonstrate that the ethanol-injection-derived Lip-LF effectively enhances intestinal retention while maintaining biocompatibility, as evidenced by the absence of organ toxicity or histological damage, thereby validating its safety and efficacy for subsequent functional studies in drug delivery applications.

### 3.5. Lip-LF Against RIII

**Figure 5:**
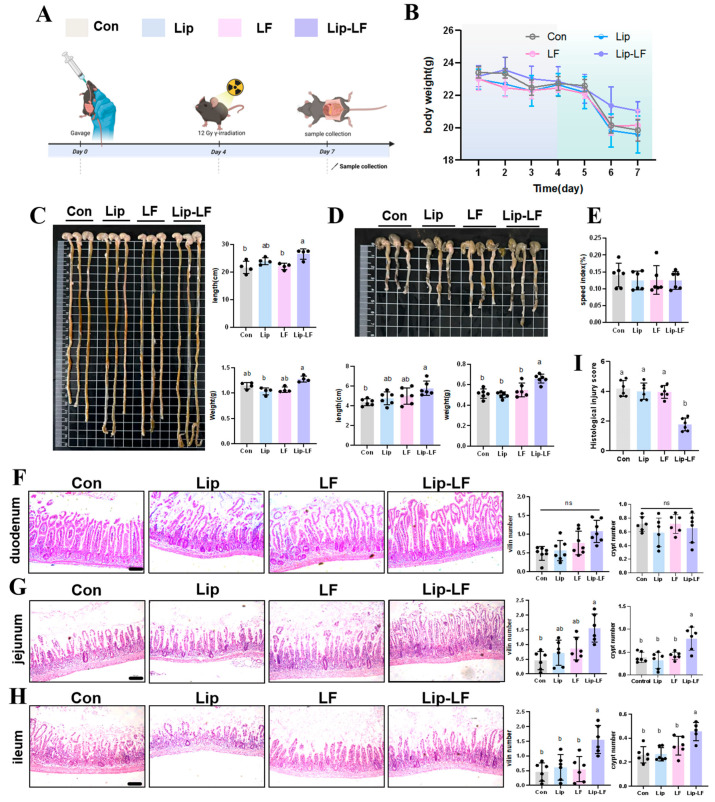
Supplement of Lip-LF mitigates IR-induced intestinal damage: (**A**) Processing and molding scheme. (**B**) Changes in mouse body weight during gavage. (**C**) Effect of con, Lip, LF, and Lip-LF on the small intestine of mice after radiation injury. (**D**) Effect of con, Lip, LF, and Lip-LF on the colon of mice after radiation injury. (**E**) Effect of con, Lip, LF, and Lip-LF on the spleen after irradiation injury. (**F**) HE staining of the duodenum. (**G**) HE staining of the ileum. (**H**) HE staining of the ileum. (**I**) Damage score of the jejunum. Con: control group, irradiation + H_2_O gavage; LF: irradiation + LF gavage; Lip: irradiation + phospholipid cholesterol liposome gavage; Lip-LF: irradiation + liposome-encapsulated lactoferrin gavage; *n* = 6). The statistical method within the group was an ANOVA test. Different letters indicated significant differences (*p* < 0.05), and those without labels indicated no within-group differences.

To investigate the in vivo functionality of Lip-LF, we employed an ionizing radiation (IR) injury model (Figure 5A). After 12 Gy IR, body weights exhibited a transient decline, with no significant differences observed between groups (Figure 5B). Notably, Lip-LF significantly preserved small-intestinal length (23.92 ± 1.23 cm vs. 21.6 ± 2.29 cm, *p* < 0.05) and weight (1.28 ± 0.06 g vs. 1.04 ± 0.06 g, *p* < 0.05) compared to the control group, whereas LF and Lip intervention remained unchanged (Figure 5C). These findings demonstrate that LF encapsulation in liposomes can promote the repair of RIII. Similar results were observed in the colon (Figure 5D). The absence of differences in the spleen organ index among all groups indicates that the liposomes are safe, non-toxic, and free of side effects (Figure 5E).

To further assess the effects of Lip-LF on intestinal tissue post-injury, H&E staining was conducted. As shown in Figure 5F, the duodenum exhibited intact villi and crypts, with no differences among groups, indicating no impact from gavage treatments. The jejunum of the control group exhibited severe pathological damage, with complete destruction of the villus structure and disordered crypt structure. Although the LF gavage group showed some improvement, the intestinal villi still had obvious damage, and the repair effect was limited. In contrast, the Lip-LF group maintained intact villus structure and demonstrated significant tissue repair effects (Figure 5G). Statistical results indicated that the number of crypts and intact villi in the jejunum of the Lip-LF group was significantly higher than that of the LF, Lip, and control groups (Figure 5G). Furthermore, as shown in Figure 5H, the Lip-LF group had significantly more intact villi in the ileum (*p* < 0.05), suggesting enhanced IEC proliferation and differentiation, which contributed to villus integrity and ileal tissue protection. Relative to the treatment of PBS, administration of Lip-LF substantially alleviated the jejunum damage (Figure 5I), reducing the tissue damage score of the intestine in IR mice. This study highlights the efficacy of Lip-LF in mitigating RIII while ensuring safety and minimizing adverse effects.

### 3.6. Lip-LF Promote the Proliferation and Goblet-Cell Differentiation

**Figure 6:**
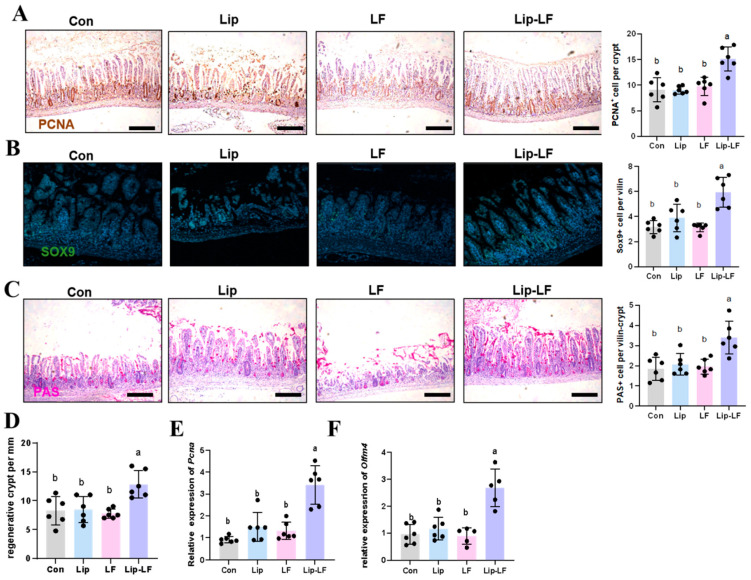
Lip-LFs promote the proliferation and differentiation of small intestinal epithelium after radiation injury: (**A**) Image of PCNA immunohistochemical staining in irradiated small intestine with con, Lip, LF, and Lip-LF treatment. (**B**) Image of immunofluorescence staining of Sox9 in irradiated small intestine with con, Lip, LF, and Lip-LF treatment. (**C**) Image of PAS staining of Sox9 in irradiated small intestine with con, Lip, LF, and Lip-LF treatment. (**D**) Results of regenerative crypt in irradiated small intestine with con, Lip, LF, and Lip-LF treatment. (**E**,**F**) Quantitative analysis of mRNA genes of Pcna and Olfm4 in the small intestine of mice under different gavage treatments. Con: control group, irradiation + H_2_O gavage; LF: irradiation + LF gavage; Lip: irradiation + phospholipid cholesterol liposome gavage; Lip-LF: irradiation + liposome-encapsulated lactoferrin gavage; *n* = 6). The statistical method within the group was ANOVA test. Different letters indicated significant differences (*p* < 0.05), and those without labels indicated no within-group differences. scale bar = 100 μm.

Intestinal stem cells (ISCs) in the small intestinal crypts are highly sensitive to irradiation and are prone to damage, which can lead to villus shortening and injury [28]. To further evaluate how LF affects intestinal epithelial outcomes and promotes repair after injury, we assessed the proliferative and differentiation levels in the jejunum. PCNA plays a crucial role in the proliferation of ISCs and DNA damage repair, and its expression levels directly reflect the proliferative state of these cells, providing an important basis for assessing the degree of intestinal injury repair. Immunohistochemical analysis revealed a significant increase in PCNA-positive signals in the Lip-LF group compared with the control group, indicating that Lip-LF promotes proliferation and repair in RIII (Figure 6A,D). The QPCR result further demonstrated upregulation of *Pcna* and *Olfm4* (Figure 6E,F), corroborating enhanced epithelial regeneration. Stem/progenitor cells are essential for the self-renewal of the small intestinal epithelium and serve as a vital source of differentiated cells. Sox9 is a marker gene for small intestinal epithelial stem/progenitor cells. Immunofluorescence showed elevated Sox9+ stem/progenitor cells within Lip-LF villi (Figure 6B), substantiating the proliferative advantage. PAS staining disclosed a significant rise in goblet-cell density within Lip-LF villi (Figure 6C), indicating reinforced mucus barrier function. Collectively, these data support that Lip-LF not only accelerates epithelial proliferation but also drives terminal differentiation, restoring intestinal homeostasis after radiation insult.

## 4. Discussion

RIII remains a dose-limiting toxicity of abdominopelvic radiotherapy that lacks effective interventions. In this study, we demonstrate that liposomal encapsulation converts LF into an intestine-targeted nanotherapy capable of accelerating epithelial regeneration after lethal irradiation. The observed benefits originate from the synergistic integration of three design principles: gastric protection and controlled intestinal release, preferential accumulation within the injured mucosa, and multi-modal activation of crypt stem-cell proliferation and differentiation.

LF has been identified as a promising agent for maintaining intestinal barrier function and mitigating inflammatory responses, as evidenced by its ability to modulate key cellular pathways and molecular mechanisms. In vitro studies demonstrate that LF promotes intestinal epithelium by downregulating ROS and pro-inflammatory cytokines while restoring tight-junction proteins (Claudin-1, Occludin, ZO-1) and TEER [12,29,30,31]. This protective effect is mediated through suppression of MAPK/NF-κB and activation of PI3K/Akt–Nrf2 pathways, reducing apoptosis and preserving barrier integrity [29,31]. Also, LF has repeatedly been shown to accelerate crypt regeneration, restore epithelial integrity, and suppress inflammation in LPS- or DSS-induced colitis [30,31,32]. However, given that native LF is rapidly degraded by gastrointestinal enzymes [33], rapidly hydrolyzed at gastric pH [34], and structural integrity is crucial for its multifunctional properties [35,36], its application is significantly limited. Advanced encapsulation technologies, such as enteric coatings, liposomes, and nanofibers, are essential for LF activity and optimizing its intestinal delivery, particularly to concentrate it at the irradiated intestine [37].

Using the ethanol-injection method, we successfully constructed LF-loaded liposomes with a Chol: EYPC ratio of 2:8, a LF concentration of 12.5 mg mL^−1^, a drug-loading capacity of 6.8%, and an encapsulation efficiency of >60%. These parameters are comparable to those reported in previous LF-encapsulated systems [38,39]. The liposomes exhibited a zeta potential of −38.03 ± 5.70 mV, ensuring colloidal stability during 6-day storage at 4 °C. Lip-LF demonstrated superior performance compared to free LF in terms of stability and release. It retained 80% protein integrity after 120 min in simulated gastric fluid, attributed to its impermeable lipid bilayer. Furthermore, Lip-LF released LF in the jejunum through bile salt-mediated solubilization. This pH-responsive behavior minimizes systemic exposure while enhancing local efficacy, which is critical for treating RIII. Real-time fluorescence imaging revealed that Lip-LF’s prolonged jejunal residence, due to its negative surface charge and nanoscale dimensions, is essential for targeting the radiosensitive jejunum. This finding aligns with reports that anionic liposomes can extend transit time by more than twofold [40]. Other LF encapsulation methods have been explored, such as using microbial transglutaminase to cross-link LF–α-lactalbumin complexes [41], developing pectin–chitosan-modified solid lipid nanoparticles (LF-SLNs) for enhanced uptake and permeability [38], embedding iron-saturated LF in alginate–chitosan microbeads for slow release [39], and creating Eudragit S100 nanofibers for colonic delivery [42]. However, existing LF carriers face challenges in stability, controlled release, or food compatibility. Our liposomal system overcomes these limitations through food-grade encapsulation, gastric protection, and enhanced intestinal targeting, offering a clinically viable solution for LF delivery. The Lip-LF formulation, with its pH-responsive release and prolonged jejunal residence, uniquely targets the small intestine in RIII. It combines high drug-loading capacity, colloidal stability, and prolonged exposure, making it a promising candidate for LF delivery. Future work will focus on preclinical efficacy evaluation.

LF exerts multifaceted beneficial effects on gut health by reducing oxidative stress and inflammation, promoting tissue repair, and enhancing barrier integrity through mechanisms such as preserving villus height and tight-junction protein expression [32,43]. It also stimulates crypt cell proliferation and differentiation via Wnt/β-catenin and PI3K/Akt-ERK pathways, supports microbiota modulation, and boosts short-chain fatty acid production [44,45]. Additionally, LF suppresses NF-κB while activating PPAR signaling to mitigate LPS-induced inflammation and preserve barrier function [46]. In our study, Lip-LF demonstrated superior efficacy in a lethal irradiation model, restoring villus height, increasing PCNA/Sox9/Olfm4-positive crypt stem cells, and enhancing goblet cell density, whereas free LF or empty liposomes did not. This aligns with previous findings showing LF-induced activation of Wnt/β-catenin and PI3K/Akt pathways [44,47,48] and radioprotection via reduced oxidative stress and inflammation [43]. Collectively, these findings suggest that Lip-LF represents a promising targeted delivery system for maximizing the radioprotective and intestinal regenerative potential of LF, warranting further investigation into its translational applications for radiation-induced gut injury and inflammatory bowel diseases. The enhanced efficacy of Lip-LF may stem from its ability to achieve sustained mucosal delivery and localized bioavailability, which synergistically amplifies the activation of Wnt/β-catenin and PI3K/Akt-ERK pathways while concurrently suppressing pro-inflammatory NF-κB signaling, creating a multi-pronged protective cascade against RIII. Collectively, these findings suggest that Lip-LF represents a promising targeted delivery system for maximizing the radioprotective and intestinal regenerative potential of LF, warranting further investigation into its translational applications for RIII and inflammatory bowel diseases.

Although Lip-LF shows great potential in the intervention of radiation enteropathy, there are still some limitations in the current research. This study provides histological and molecular evidence for the radioprotective effects of Lip-LF, though direct functional assessment of intestinal barrier integrity (e.g., FITC–dextran permeability) is not included. Future studies will incorporate functional metrics to further validate barrier restoration and targeting efficacy. The murine model employed a single-dose, single-sex design, failing to fully recapitulate human radiotherapy regimens, which typically involve fractionated dosing and concurrent chemotherapy. Additionally, the interactions between Lip-LF and the gut microbiota, a critical factor in RIII, remain unexplored, necessitating future metagenomic sequencing to elucidate microbial modulation. The liposomal formulation also requires further optimization to enhance targeting, biodistribution, and long-term safety, with surface modifications and scalable microfluidic production being key areas for improvement. Moving forward, studies should incorporate advanced preclinical models (e.g., large animals with fractionated radiation), investigate microbiome-mediated mechanisms, and explore combination therapies (e.g., probiotics or SCFAs) to maximize therapeutic efficacy. Ultimately, clinical translation will depend on rigorous evaluation of Lip-LF in human trials, ensuring both safety and therapeutic potential in radiation enteropathy patients. Addressing these gaps will pave the way for more effective, clinically viable interventions.

## 5. Conclusions

Here, we demonstrate that LF encapsulated in anionic liposomes (Lip-LF) serves as a potent, gut-targeted nanotherapy for RIII. By optimizing the lipid composition (Chol: EYPC = 2:8, *w*/*w*) and drug-loading concentration (12.5 mg mL^−1^), we achieved a narrow size distribution and excellent storage stability. In vitro and in vivo studies showed that Lip-LF protects LF from gastric degradation, enables pH-triggered release in the intestinal lumen, and preferentially accumulates at damaged mucosa. Functionally, Lip-LF treatment restored villus counts, increased crypt height, promoted goblet-cell regeneration, and enhanced epithelial proliferation (PCNA/Sox9/Olfm4 upregulation) with negligible systemic toxicity. This study establishes Lip-LF as a biocompatible nano-platform that enhances LF’s radioprotective effects through precise intestinal delivery, offering a promising strategy for managing oncological radiotherapy complications.

## Figures and Tables

**Table 1:** Primer sequence for real-time fluorescent quantitative PCR.

Gene	Forward Primer 5′–3′	Reverse Primer 5′–3′
*Actb*	GGCTGTATTCCCCTCCATCG	CCAGTTGGTAACAATGCCATGT
*Olfm4*	CAGCCACTTTCCAATTTCACTG	GCTGGACATACTCCTTCACCTTA
*Pcna*	TTTGAGGCACGCCTGATCC	GGAGACGTGAGACGAGTCCAT

## Data Availability

The original contributions presented in this study are included in the article. Further inquiries can be directed to the corresponding author.
